# Interferon Signaling Is Frequently Downregulated in Melanoma

**DOI:** 10.3389/fimmu.2018.01414

**Published:** 2018-06-21

**Authors:** Sara Alavi, Ashleigh Jacqueline Stewart, Richard F. Kefford, Su Yin Lim, Elena Shklovskaya, Helen Rizos

**Affiliations:** ^1^Department of Biomedical Sciences, Faculty of Medicine and Health Sciences, Macquarie University, Sydney, NSW, Australia; ^2^Melanoma Institute Australia, Sydney, NSW, Australia; ^3^Department of Clinical Medicine, Faculty of Medicine and Health Sciences, Macquarie University, Sydney, NSW, Australia

**Keywords:** melanoma, interferon, antigen presentation, immunosuppression, PD-L1, PD-L2

## Abstract

Immune checkpoint inhibitors that block the programmed cell death protein 1/PD-L1 pathway have significantly improved the survival of patients with advanced melanoma. Immunotherapies are only effective in 15–40% of melanoma patients and resistance is associated with defects in antigen presentation and interferon signaling pathways. In this study, we examined interferon-γ (IFNγ) responses in a large panel of immune checkpoint inhibitor-naïve melanoma cells with defined genetic drivers; *BRAF*-mutant (*n* = 11), *NRAS*-mutant (*n* = 10), *BRAF/NRAS* wild type (*n* = 10), and *GNAQ/GNA11*-mutant uveal melanomas (UVMs) (*n* = 8). Cell surface expression of established IFNγ downstream targets PD-L1, PD-L2, HLA-A, -B, and -C, HLA-DR, and nerve growth factor receptor (NGFR) were analyzed by flow cytometry. Basal cellular expression levels of HLA-A, -B, -C, HLA-DR, NGFR, and PD-L2 predicted the levels of IFNγ-stimulation, whereas PD-L1 induction was independent of basal expression levels. Only 13/39 (33%) of the melanoma cell lines tested responded to IFNγ with potent induction of all targets, indicating that downregulation of IFNγ signaling is common in melanoma. In addition, we identified two well-recognized mechanisms of immunotherapy resistance, the loss of β-2-microglobulin and interferon gamma receptor 1 expression. We also examined the influence of melanoma driver oncogenes on IFNγ signaling and our data suggest that UVM have diminished capacity to respond to IFNγ, with lower induced expression of several targets, consistent with the disappointing response of UVM to immunotherapies. Our results demonstrate that melanoma responses to IFNγ are heterogeneous, frequently downregulated in immune checkpoint inhibitor-naïve melanoma and potentially predictive of response to immunotherapy.

## Introduction

The identification of checkpoint signaling pathways that dampen anti-cancer immune responses and the subsequent development of checkpoint inhibitors have transformed the treatment of patients with metastatic cancer. Antibodies blocking immune checkpoints such as the cytotoxic T-lymphocyte-associated protein-4, the programmed cell death protein 1 (PD-1), and its ligand PD-L1 induce durable anti-tumor immune responses in many advanced malignancies, including melanoma, non-small-cell lung cancer, and renal cell carcinoma. PD-1 inhibition in melanoma promotes tumor regression and prolonged overall survival in 30–40% of patients with advanced disease (1–3). However, the majority of melanoma patients will not benefit from immunotherapy and 25% of responding patients will relapse within 2 years (4).

Recent studies have shown that resistance to immune checkpoint blockade involves defects in the interferon-γ (IFNγ) signaling pathway (5–9). Once secreted by activated T cells, IFNγ binds and activates the IFNγ receptor complex (IFNGR1/2), which is broadly expressed on many cell types, including cancer cells. Receptor binding leads to the activation of the receptor-associated Janus kinases (JAK1 and 2) which phosphorylate and activate the signal transducer and activator of transcription (STAT) proteins, STAT1 and STAT3. Nuclear translocation of STAT transcription factors promotes the transcription of hundreds of IFNγ response genes (10) including downstream transcription factors, such as IRF1, STAT1, and STAT3, genes involved in antigen presentation such as MHC class I and II molecules (8, 11), and genes that attenuate immune activity to minimize local tissue damage, such as PD-L1 and PD-L2 (7). The multifunctional effects of IFNγ are particularly important in the context of immunotherapy since enhanced antigen presentation improves immune recognition of tumors while expression of immunosuppressive molecules limits anti-tumor T cell activity.

Several genetic defects affecting the IFNγ signaling pathway are associated with melanoma resistance to immunotherapy, including checkpoint inhibition. For instance, the genetic loss of the β-2-microglobulin (*B2M*) gene, the structural component of MHC class I complexes, is enriched in pre-treatment tumor samples from melanoma patients with innate and acquired resistance to checkpoint inhibitor therapy (12, 13). Genetic alterations affecting *IFNGR1, IFNGR2, IRF1*, and *JAK2*, and amplifications of the IFNγ inhibitor genes, *SOCS1* and *PIAS4*, are also enriched in patients not responding to checkpoint inhibition (6). Furthermore, loss-of-function mutations in the upstream IFNγ-signaling regulators *JAK1* and *JAK2*, concurrent with deletion of the wild type alleles, have been identified in two melanoma patients who failed anti-PD-1 therapy (7). The loss of IFNγ signaling limits immune cell recruitment and immune recognition of tumor cells by suppressing the production of IFNγ-dependent chemokines and diminishing antigen presentation (8, 9, 14).

In this study, we investigated the response of a large panel of human melanoma cells to IFNγ stimulation. These cells were naïve to immune checkpoint inhibitors, and we examined whether the expression of key IFNγ downstream targets [PD-L1, PD-L2, nerve growth factor receptor (NGFR), HLA-A, -B, -C, and HLA-DR] could serve to assess the integrity of IFNγ signaling in melanoma. We also examined the potential influence of melanoma driver oncogenes on IFNγ signaling activity and found that uveal melanoma (UVM) cells show evidence of diminished IFNγ pathway activity with minimal baseline and IFNγ induction of HLA-DR, NGFR, and PD-L2. Importantly, nearly 70% of melanoma cells included in this study showed incomplete responses to IFNγ stimulation, indicative of pre-existing resistance to immunotherapy. Furthermore, our data confirm that measuring IFNγ output with a select number of targets may be useful for detecting intrinsic defects in the IFNγ/JAK/STAT pathway, including JAK and STAT mutations which are associated with PD-1 inhibitor resistance (7, 8, 13).

## Materials and Methods

### Cell Lines

A total of 39 cell lines were included in this study. Oncogenic driver mutation status is shown in Table 1. Melanoma cell lines were provided by Prof. Nicholas Hayward and Prof. Peter Parsons at QIMR Berghofer Medical Research Institute, Australia, Prof. Bruce Ksander at Harvard Medical School, MA, Prof. Peter Hersey at the Centenary Institute, Sydney, Australia, and Prof. Xu Dong Zhang at the University of Newcastle, Newcastle, Australia. Two short-term melanoma cell lines were cultured from surgically excised, enzymatically processed melanoma lesions (SCC14-0257, SMU15-0217) in a study carried out in accordance with the recommendations of Human Research ethics committee protocols from Royal Prince Alfred Hospital (Protocol X15-0454 and HREC/11/RPAH/444). Cell authentication was confirmed using the StemElite ID system from Promega.

**Table 1:** Expression of IFNγ-target proteins at baseline and post-stimulation with IFNγ in 39 melanoma cell lines.

Cell line	Driver mutation	HLA-ABC		HLA-DR		NGFR		PD-L1		PD-L2	
		−	+	−	+	−	+	−	+	−	+
A2058	BRAF^V600E^	32.9	139.1	2.5	66.7	398.7	218.6	1.0	9.8	1.9	7.7
SKMel28	BRAF^V600E^	74.2	128.3	11.1	120.8	44.7	197.3	1.3	2.9	2.6	10.0
C060M1	BRAF^V600E^	34.9	88.7	29.5	75.7	10.4	16.3	1.0	5.5	4.3	6.7
SCC14-0257	BRAF^V600K^	15.8	64.8	12.8	102.2	347.3	722.7	0.9	2.5	1.4	5.9
MM418	BRAF^V600E^	38.2	81.8	1.2	7.7	16.0	19.0	1.1	4.0	1.1	1.5
NM16	BRAF^V600E^	21.7	62.5	7.0	76.5	1,808.8	2,833.0	0.8	3.4	7.8	17.1
NM182	BRAF^V600E^	18.9	143.6	1.3	31.8	10.1	14.0	1.0	4.5	1.0	1.6
MM200	BRAF^V600E^	35.1	202.0	7.2	141.0	450.7	1,505.6	1.0	4.0	1.2	3.4
NM39	BRAF^V600E^	43.6	122.2	27.6	123.3	90.7	46.7	1.1	3.9	4.3	17.9
HT144	BRAF^V600E^	23.4	43.4	71.4	100.3	353.1	292.9	1.1	3.2	4.9	12.0
C016M	BRAF^V600E^	20.9	70.3	42.7	95.9	469.6	630.2	0.9	1.3	2.2	5.5
MelRm	NRAS^Q61R^	36.9	98.7	102.8	252.5	18.4	75.2	0.9	2.3	1.6	3.4
NM47	NRAS^Q61R^	42.1	109.0	157.9	249.0	213.6	1,034.1	1.0	2.5	1.8	2.9
NM177	NRAS^Q61R^	56.5	99.7	76.6	92.9	3,674.9	3,663.2	1.0	1.9	2.0	3.4
NM179	NRAS^Q61K^	12.7	31.1	2.4	59.6	44.2	85.4	1.0	3.3	1.7	6.6
ME4405	NRAS^Q61R^	60.5	118.2	0.9	0.9	24.0	47.3	1.0	2.1	3.5	14.2
MelAT	NRAS^Q61R^	31.9	121.3	0.9	1.0	11.8	27.4	1.2	2.0	2.0	10.3
D11M2	NRAS^Q61L^	11.3	18.4	16.4	33.7	29.2	32.1	1.1	2.4	3.3	9.2
C002M	NRAS^Q61K^	7.2	31.4	1.0	27.0	13.7	15.6	1.2	2.3	1.4	1.5
C013M	NRAS^Q61L^	24.7	81.1	1.0	42.7	47.8	199.3	1.0	2.3	2.2	6.5
D38M2	NRAS^Q61R^	28.6	86.2	4.5	56.5	509.6	480.4	1.1	2.4	2.9	12.2
D22M1	BRAF/NRAS^WT^	28.0	25.7	1.9	1.9	5.8	5.3	1.3	1.1	1.3	1.2
MeWo	BRAF/NRAS^WT^	28.9	107.6	1.4	19.1	268.9	176.2	0.9	2.2	1.3	4.2
D24M	BRAF/NRAS^WT^	32.6	37.2	13.8	43.3	30.7	28.3	1.2	3.3	7.3	31.0
C022M1	BRAF/NRAS^WT^	10.7	41.7	2.3	21.5	148.3	341.0	1.9	1.6	1.1	1.8
C084M	BRAF/NRAS^WT^	83.6	119.0	19.5	130.4	552.5	631.5	0.9	4.6	3.2	15.7
C086M	BRAF/NRAS^WT^	20.1	52.4	22.7	90.3	1.3	2.9	1.1	3.0	3.7	9.9
D35	BRAF/NRAS^WT^	167.9	460.2	3.8	129.6	21.9	36.9	0.9	2.9	0.9	2.7
C025M1	BRAF/NRAS^WT^	73.2	134.1	2.1	18.5	1.7	2.7	1.1	2.7	1.1	1.2
SMU15-0217	BRAF/NRAS^WT^	1.5	2.1	12.8	91.4	11.0	26.0	1.2	3.4	4.4	22.3
A04-GEH	BRAF/NRAS^WT^	23.9	96.6	1.5	63.3	13.8	45.9	1.0	2.9	1.2	8.2
92.1	GNAQ^Q209L^	11.5	87.2	0.5	0.5	14.0	38.8	1.1	1.7	1.2	1.0
MEL202	GNAQ^Q209L, R210K^	38.2	346.3	1.1	15.9	10.6	15.0	1.0	5.6	1.0	2.7
MEL270	GNAQ^Q209P^	52.5	115.6	1.1	1.8	3.6	4.1	1.1	1.6	1.2	1.3
MP38	GNAQ^Q209P^	73.5	329.7	1.6	10.6	10.6	15.8	1.1	2.4	2.9	25.3
OMM1	GNA11^Q209L^	31.2	108.0	1.1	19.0	2.0	3.6	1.1	1.5	1.0	3.7
MP41	GNA11^Q209L^	26.3	44.2	0.9	3.7	2.6	3.6	0.9	1.3	1.1	2.0
MP46	GNAQ^Q209L^	2.3	38.6	1.0	1.0	5.2	10.6	1.1	1.5	1.0	1.6
MM28	GNA11^Q209L^	9.4	50.8	1.1	13.1	1.3	1.7	1.0	2.1	1.0	2.1

*Relative marker expression levels were calculated by dividing the geometric mean fluorescence intensity (MFI) of the antibody-stained sample by the FMO control MFI*.

*−, no IFNγ treatment; +, treated for 72 h with 1,000 U/ml IFNγ; IFNγ, interferon-γ; NGFR, nerve growth factor receptor; FMO, fluorescence minus one*.

### Cell Culture

Cell lines were cultured in Dulbecco’s Modified Eagle Medium or Roswell Park Memorial Institute-1640 media supplemented with 10 or 20% heat inactivated fetal bovine serum (FBS; Sigma-Aldrich, St. Louis, MO, USA), 11.25 mM glutamine (Gibco, Thermo Fisher Scientific, Waltham, MA, USA), and 10 mM HEPES (Gibco) and were maintained at 37°C in 5% CO_2_. For IFNγ treatment, 7 × 10^4^ melanoma cells per well were plated in complete media in six-well plates. After an overnight incubation, the complete media was replenished, and cells treated for 72 h with 1,000 U/ml IFNγ (Peprotech, Rocky Hill, NJ, USA) or vehicle control [0.1% bovine serum albumin (Sigma-Aldrich) in phosphate-buffered saline (PBS, Gibco)]. Cells were collected, washed with PBS, and analyzed by flow cytometry.

### Flow Cytometry

Staining was performed in flow cytometry buffer (PBS supplemented with 5% FBS, 10 mM EDTA, and 0.05% sodium azide). Cells (2 × 10^5^) were incubated for 30 min on ice with mouse anti-human antibodies against HLA-ABC (clone W6/32), HLA-DR (clone L243), CD271/NGFR (clone ME20.4), CD273/PD-L2 (clone 24F.10C12) (all from BioLegend, San Diego, CA, USA), and CD274/PD-L1 (clone MIH1; BD Biosciences, Franklin Lakes, NJ, USA) conjugated to phycoerythrin (PE), fluorescein isothiocyanate, PE-cyanine (Cy)7, allophycocyanin, and brilliant violet 421, respectively. All antibodies were titrated prior to experiment to ensure optimal concentrations were used. Fc block (BD Biosciences) was used to prevent non-specific staining due to antibody binding to Fc receptors. Fluorescence minus one controls (FMO, staining with all but one antibody for each fluorochrome) were included with each experiment. Prior to acquisition, cell viability was determined by staining cells with either 5 µM DAPI (Invitrogen, Thermo Fisher Scientific), Zombie Yellow dye (BioLegend), or Live Dead near-IR fixable dye (Invitrogen, Thermo Fisher Scientific). For the analysis of interferon gamma receptor 1 (IFNGR1) and B2M expression, cells were first stained with a fixable viability dye and either PE-conjugated anti-CD119 (clone GIR-208) or PE-Cy7 conjugated anti-B2M (clone 2M2), both from BioLegend. Cells were then fixed and permeabilized using the BD Cytofix/Cytoperm kit and stained intracellularly with the same antibody that was used for cell surface stain.

**Figure 1:**
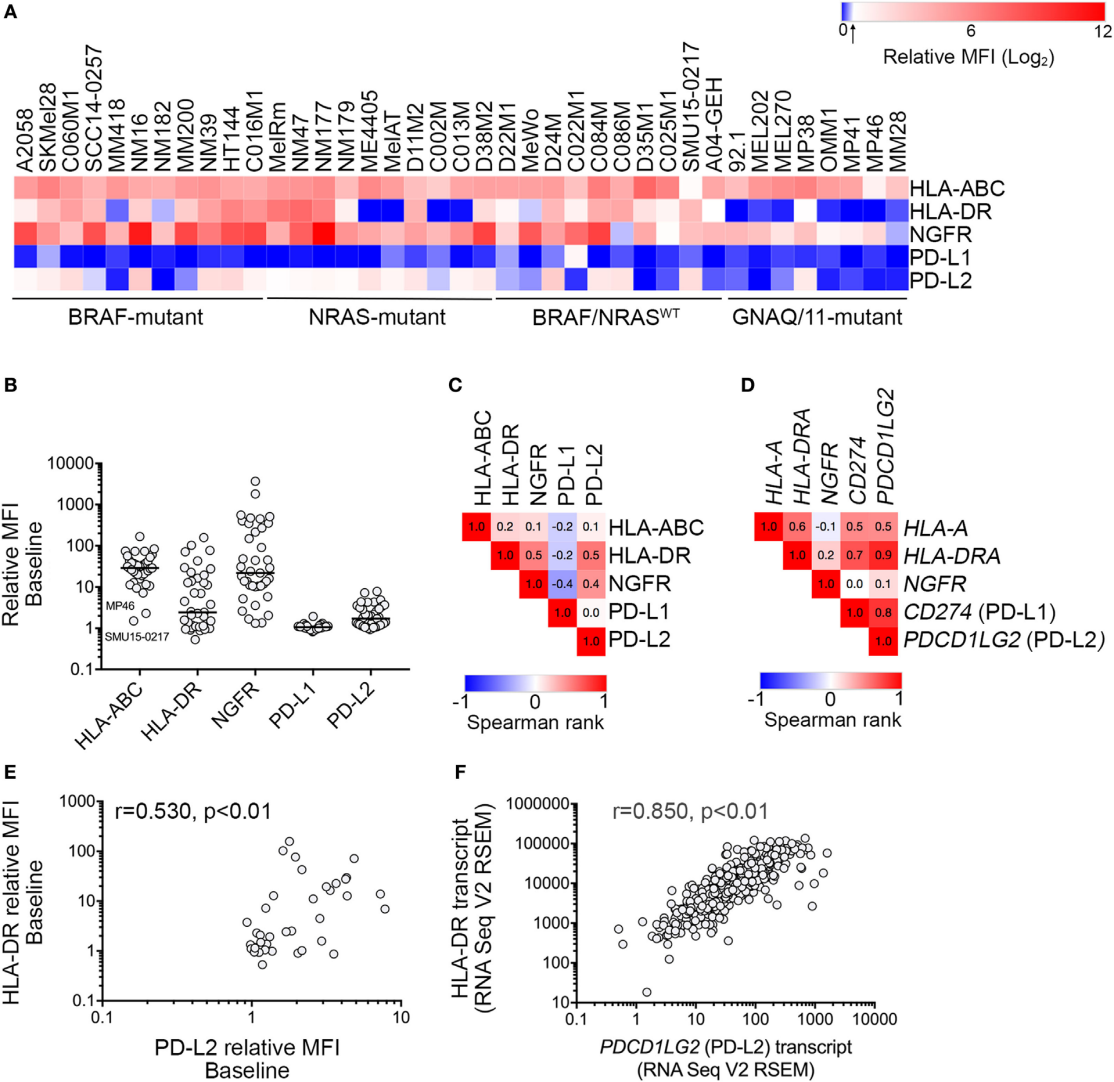
Expression of downstream interferon-γ targets in melanoma cells. **(A)** Heatmap showing cell surface expression [relative mean fluorescence intensity (MFI); mean of two to five independent experiments] of HLA-ABC, HLA-DR, nerve growth factor receptor (NGFR), PD-L1, and PD-L2 in 39 melanoma cell lines with defined oncogenic drivers including 11 *BRAF^V600^*-mutant, 10 *NRAS*-mutant, 10 *BRAF/NRAS* wild type (*BRAF/NRAS*^WT^), and 8 *GNAQ/11*-mutant uveal melanoma cell lines. Relative MFI < 1.5 is indicated by the arrow on the color bar. **(B)** Cell surface baseline expression (relative MFI) of HLA-ABC, HLA-DR, NGFR, PD-L1, and PD-L2 in a panel of 39 melanoma cell lines. Each dot represents one cell line and the median expression is indicated by the horizontal line. Low cell surface expression of HLA-ABC on the MP46 and SMU15-0217 cell lines is indicated. **(C)** Correlation matrix showing Spearman’s rank correlation analysis between cell surface expression of markers, as indicated. Spearman’s rank correlation values are shown within the similarity matrix. **(D)** Correlation matrix showing Spearman’s rank correlation analysis between transcript levels of *HLA-A, HLA-DRA, NGFR, PD-L1*, and *PD-L2* (The Cancer Genome Atlas (TCGA) skin cutaneous melanoma (SKCM) dataset). Spearman’s rank correlation is shown within the similarity matrix. **(E)** Correlation between PD-L2 and HLA-DR cell surface expression and **(F)** mRNA transcript expression (TCGA SKCM dataset). Spearman’s rank correlation coefficient and *p* values are shown.

Samples were acquired on BD LSRFortessa X20 flow cytometer (BD Biosciences) and the FlowJo software (TreeStar, Ashland, OR, USA) was used for data analysis. At least 10,000 live events were acquired. General gating strategy included forward and side scatter area to exclude cell debris, time parameter to exclude electronic noise, forward scatter area and height to exclude doublets and gating on viable cells (by gating on DAPI, Zombie Yellow, or Live Dead near-IR negative events). Relative marker expression levels were calculated by dividing the geometric mean fluorescence intensity (MFI) of the antibody-stained sample by the FMO control MFI (Figure 1A). Relative MFI is used in all analyses, and a relative MFI < 1.5 was considered to reflect no antigen expression relative to the control.

### Cell Cycle and Apoptosis Analysis

Adherent and floating cells were combined after 72 h treatment with vehicle control or 1,000 U/ml IFNγ and cell cycle analyses were performed as previously described (15) using at least three biological replicates.

### Gene Set Enrichment Transcriptome Analysis

Transcriptome analysis was performed on the The Cancer Genome Atlas (TCGA) human skin cutaneous melanoma (SKCM) and UVM datasets using single sample gene set enrichment analysis (ssGSEA) (16). RNA counts were normalized using the weighted trimmed mean of M-values implemented in the edgeR Bioconductor package. Normalized counts were transformed using *voom*, as implemented in the *Limma* package (17, 18). The gene sets used in ssGSEA analysis consisted of the Hallmark gene set version 6.1, a refined gene set that define specific biological processes (19).

### Whole Exome Sequencing

Melanoma cell exome sequencing was performed on D22M1 and SMU15-0217 melanoma cell lines. Exonic DNA was enriched using the Illumina SureSelect technology, targeting 50 Mb encompassing protein-coding regions and sequenced on an Illumina HiSeq2000. Read pairs were aligned to the reference human genome (hg19) using BWA (20) and nucleotide variants (SNVs) and small insertion/deletions were detected by SAMTools (21). Ingenuity Variant Analysis (http://www.ingenuity.com) was used to identify mutations in genes associated with the JAK-STAT (KEGG) signaling pathway (22).

### Statistical Analysis

Statistical significance was calculated using GraphPad Prism version 7 (GraphPad software, San Diego, CA, USA). *p*-Values <0.05 were considered significant.

## Results

### Baseline Expression of IFNγ Target Molecules in Melanoma Lines With Different Oncogenic Driver Mutations

Expression of five well-defined IFNγ targets, the PD-1 ligands PD-L1 and PD-L2, NGFR, antigen-presenting HLA-A, -B, and -C (HLA-ABC), and HLA-DR molecules was examined in a panel of 39 human melanoma cell lines with defined oncogenic driver mutations (Figure 1A; Figure S1 in Supplementary Material). These included 11 *BRAF^V600^*-mutant, 10 *NRAS*-mutant and 10 *BRAF/NRAS* wild type (*BRAF/NRAS*^WT^) cutaneous melanoma cell lines, and 8 *GNAQ/11*-mutant UVM cell lines (Table 1).

Analysis of cell surface marker expression (antibody-stained MFI/FMO control MFI, relative MFI) revealed a broad range of expression for all five markers (Figure 1; Table 1). MHC class I molecules (HLA-ABC) were uniformly expressed on melanoma cells with the exception of the *BRAF/NRAS^WT^* SMU15-0217 (relative MFI = 1.5) and the uveal MP46 cells (relative MFI = 2.3) (Figure 1B). HLA-DR showed a broad range of baseline expression in our panel of melanoma cells with no expression in 14 melanoma cell lines (MFI ratio < 1.5) and bimodal expression in 11/39 cell lines [i.e., only a proportion of cells (18–88%) expressed the marker]. NGFR expression was similarly variable (Figure 1B) with no expression at baseline in two cell lines (relative MFI < 1.5; Table 1). Similar to HLA-DR, NGFR was distributed in a bimodal fashion in six samples, with 42–81% cells expressing the marker. Three cell lines, the *BRAF^V600^*-mutant C060M1 and *BRAF/NRAS^WT^* D24M and SMU15-0217, had a bimodal expression of both HLA-DR and NGFR (data not shown). PD-1 ligands PD-L1 and PD-L2 were expressed at comparably low levels in our panel of melanoma cells (Table 1), with PD-L1 not constitutively expressed in 38/39 (relative MFI < 1.5) and PD-L2 absent in 18/39 cell lines. Seventeen melanoma lines lacked both PD-L1 and PD-L2 basal expression, including 5/10 (50%) *BRAF/NRAS^WT^*, 4/11 (36%) BRAF^V600^-mutant, 1/10 (10%) NRAS-mutant, and 7/8 (87.5%) uveal cell lines (Figure 1).

Of the targets analyzed, cell surface expression of PD-L2 was correlated with HLA-DR (Spearman’s rank 0.530, *p* < 0.01) and NGFR expression (Spearman’s rank 0.418, *p* < 0.01) (Figure 1C). The expression of HLA-DR and NGFR was also correlated (Spearman’s rank 0.497, *p* < 0.01). The cell surface protein expression patterns of these markers in our melanoma panel did not precisely reflect their transcript expression patterns in the human SKCM dataset of TCGA (*n* = 472; Figure 1D), although both protein and transcript expression of PD-L2 (*PDCD1LG2*) and HLA-DR (*HLA-DRA*) were correlated (Figures 1E,F). It is also worth noting that PD-L1 (*CD274*) and PD-L2 (*PDCD1LG2*) transcripts were correlated (Spearman’s rank = 0.793 *p* < 0.01) in the TCGA SKCM dataset, although we did not observe any correlation in their cell surface protein expression (Figure S2 in Supplementary Material).

**Figure 2:**
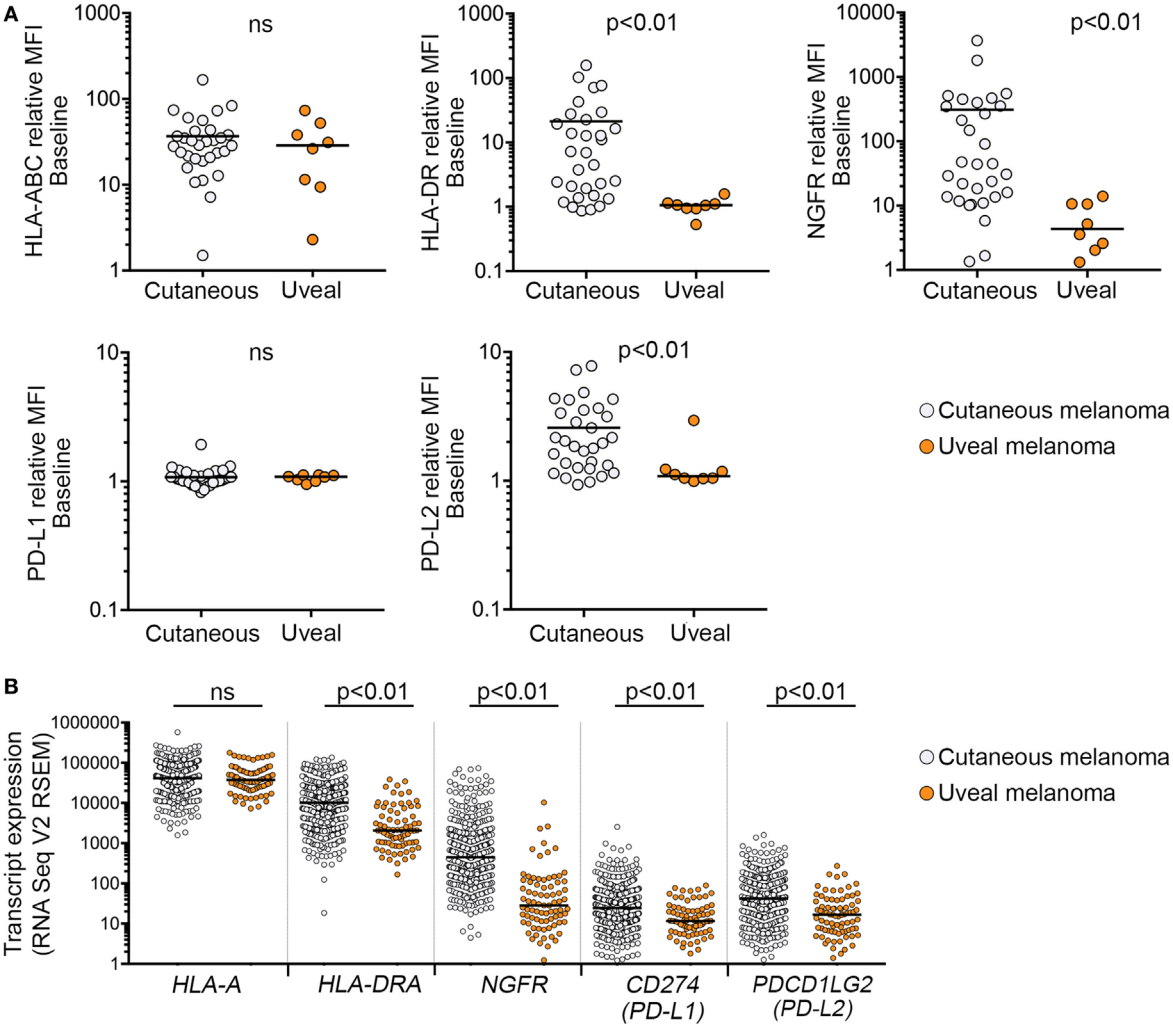
Expression of interferon-γ targets in cutaneous and uveal melanoma (UVM) cells. **(A)** Cell surface expression [relative mean fluorescence intensity (MFI)] of HLA-ABC, HLA-DR, nerve growth factor receptor (NGFR), PD-L1, and PD-L2 in cutaneous (*n* = 31) and UVM (*n* = 8) cell lines. **(B)** Expression of mRNA transcripts for *HLA-A, HLA-DRA, NGFR, PD-L1*, and *PD-L2* in the 80 uveal [The Cancer Genome Atlas (TCGA) UVM dataset] and 472 cutaneous melanoma samples (TCGA skin cutaneous melanoma dataset). Each dot represents a single sample, with the median indicated by the horizontal line. Expression levels were compared using a Mann–Whitney test; ns, not significant.

There was also evidence that basal marker expression in *GNAQ/11*-mutant UVM was distinct. In particular, HLA-DR, NGFR, and PD-L2 cell surface expression was significantly lower in the UVM cell subset compared to cutaneous melanoma (Table 1; Figure 2). To address the significance of these findings, we analyzed TCGA RNA sequencing data from 80 uveal and 472 cutaneous melanoma samples. Consistent with our cell surface expression data, the expression of *HLA-DRA, NGFR*, and PD-L2 transcripts was significantly lower in the 80 uveal compared to the 472 cutaneous melanoma samples from the TCGA dataset; *CD274* (PD-L1) transcript expression was also different between the TCGA uveal and cutaneous datasets, whereas *HLA-A* transcript expression was indistinguishable between the TCGA uveal and cutaneous tumor groups (Figure 2B).

### Expression of Target Molecules After Exposure to IFNγ

**Figure 3:**
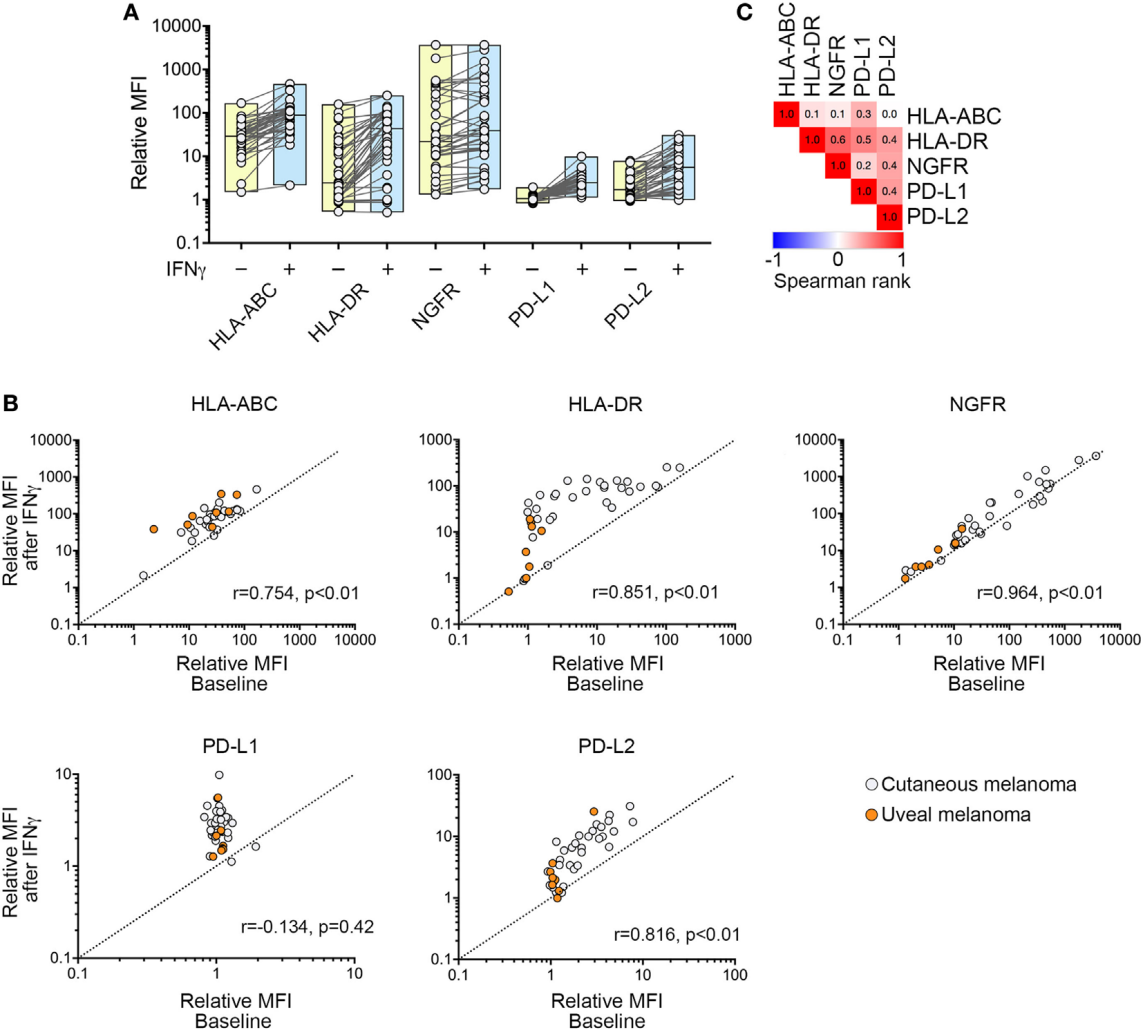
Expression of cell surface markers in response to interferon-γ (IFNγ) treatment. **(A)** Change in HLA-ABC, HLA-DR, nerve growth factor receptor (NGFR), PD-L1, and PD-L2 cell surface expression [relative mean fluorescence intensity (MFI)] after exposure to IFNγ. Each dot shows one cell line before (−) and after (+) IFNγ stimulation with box plots showing the range and median. **(B)** Correlation of baseline and IFNγ-induced cell surface expression of HLA-ABC, HLA-DR, NGFR, PD-L1, and PD-L2. Each dot represents one cell line. Spearman’s rank correlation coefficient and *p* values are shown. **(C)** Correlation matrix showing Spearman’s rank correlation analysis between IFNγ-induced expression of HLA-ABC, HLA-DR, NGFR, PD-L1, and PD-L2. Spearman rank correlation values are shown within the similarity matrix.

We noted that IFNγ stimulated the expression of HLA-ABC, HLA-DR, NGFR, PD-L1, and/or PD-L2 in the majority of melanoma cell lines (Figure 3A). The degree of IFNγ stimulation was highly variable, however, and in the case of HLA-ABC, HLA-DR, PD-L2, and NGFR, the level of stimulation was proportional to the basal expression levels (Figure 3B). Only IFNγ-induced PD-L1 expression was independent of its basal expression levels and all but four cell lines lacking baseline PD-L1 showed IFNγ-stimulation of PD-L1 expression (Figure 3B).

Comparison of all five target molecules also showed positive correlation between IFNγ-induced expression of PD-L1, PD-L2, and HLA-DR. In particular, post-stimulation levels of PD-L1 and PD-L2 were correlated (Spearman’s rank = 0.388, *p* = 0.01) (Figure 3C), although the degree of induction (i.e., change from pre- to post-stimulation) was not correlated (Spearman’s correlation = 0.315, *p* = 0.05) because PD-L1 and PD-L2 showed disparate expression at baseline (Figure 1C). Similarly, although post-stimulation levels of NGFR were correlated with induced levels of PD-L2 (Spearman’s rank = 0.358; *p* = 0.025) (Figure 3C), the degree of NGFR and PD-L2 induction (i.e., change from pre- to post-stimulation) was not correlated (Spearman’s rank = −0.103; *p* = 0.99).

**Figure 4:**
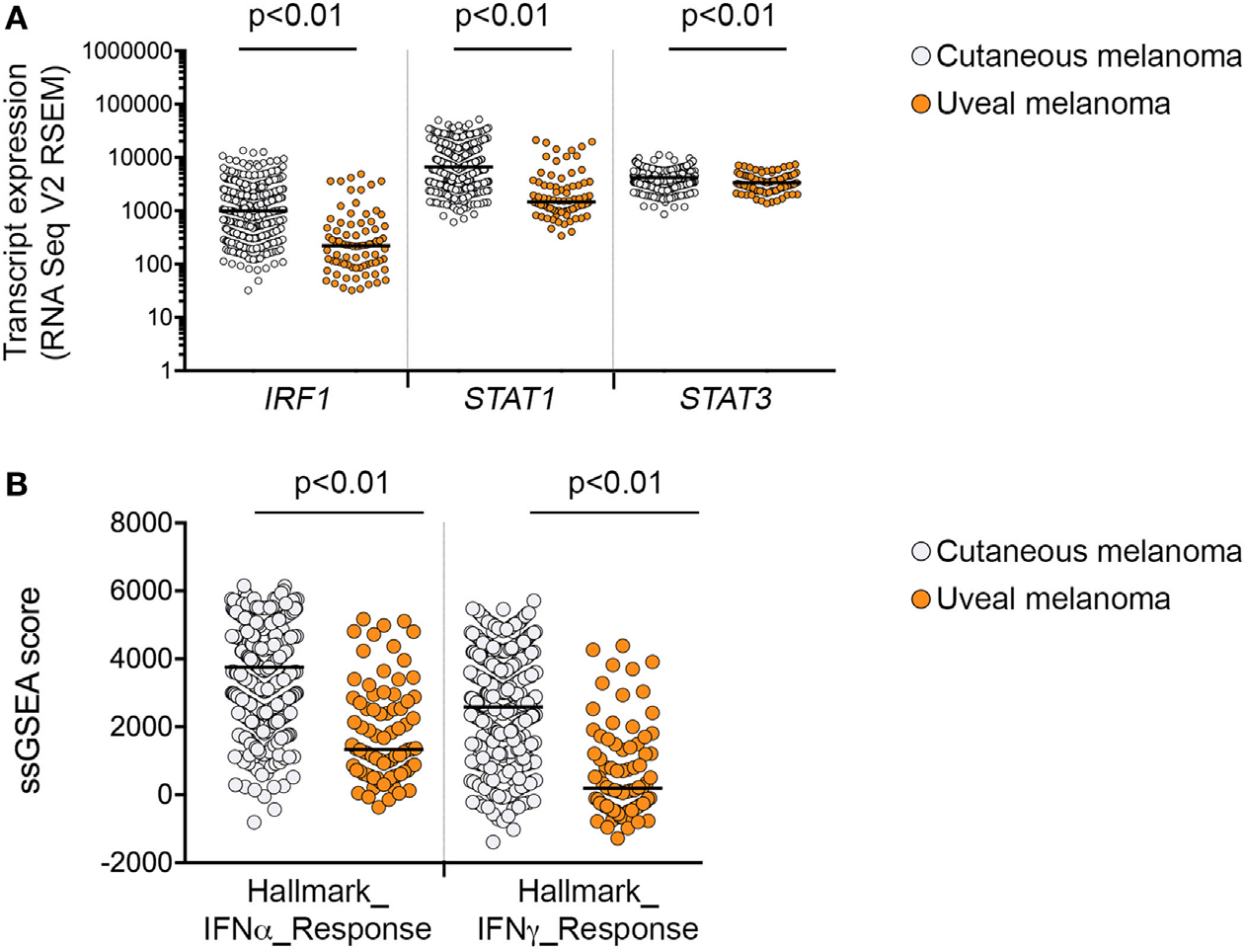
Interferon-γ signaling in cutaneous and uveal melanoma (UVM). **(A)** Expression of mRNA transcripts for *IRF1, STAT1*, and *STAT3* in the 80 uveal [The Cancer Genome Atlas (TCGA) UVM dataset] and 472 cutaneous melanoma samples (TCGA skin cutaneous melanoma dataset). **(B)** Single sample gene set enrichment analysis (ssGSEA) scores for the Hallmark_interferon_alpha and Hallmark_interferon_gamma response signatures in the 80 uveal and 472 cutaneous melanoma samples from the TCGA datasets. Expression levels were compared using a nonparametric Mann–Whitney test, *p* values are indicated.

Overall, exposure of melanoma cells to IFNγ induced heterogeneous levels of all target molecules, and induction did not appear to depend on genotype in cutaneous melanomas for PD-L1, PD-L2, HLA-ABC, and NGFR (Table 1). In UVM lines, however, the protein expression of HLA-DR, NGFR, PD-L1, and PD-L2 post-IFNγ stimulation was significantly lower than observed in cutaneous melanomas (Figure 3B; Figure S3 in Supplementary Material), and this was consistent with low baseline expression of HLA-DR, NGFR, and PD-L2 in the UVM cells (Figure 2A). The transcript expression of *STAT1, STAT3*, and *IRF1*, three key transcription factors of the IFNγ signaling cascade, were also lower in the TCGA UVM dataset compared to the TCGA cutaneous melanomas (Figure 4). We also explored interferon signaling pathways in the SKCM and uveal TCGA melanoma dataset using single sample gene set enrichment analysis (ssGSEA), an extension of GSEA that defines an enrichment score of a gene set for each of the sample in the dataset (16). As shown in Figure 4B, the enrichment scores generated for the Hallmark_interferon_alpha and Hallmark_interferon_gamma response signatures were significantly lower in the UVM dataset, compared to cutaneous melanoma.

### Downregulated Response to IFNγ in a Small Subset of Melanoma Cell Lines

**Figure 5:**
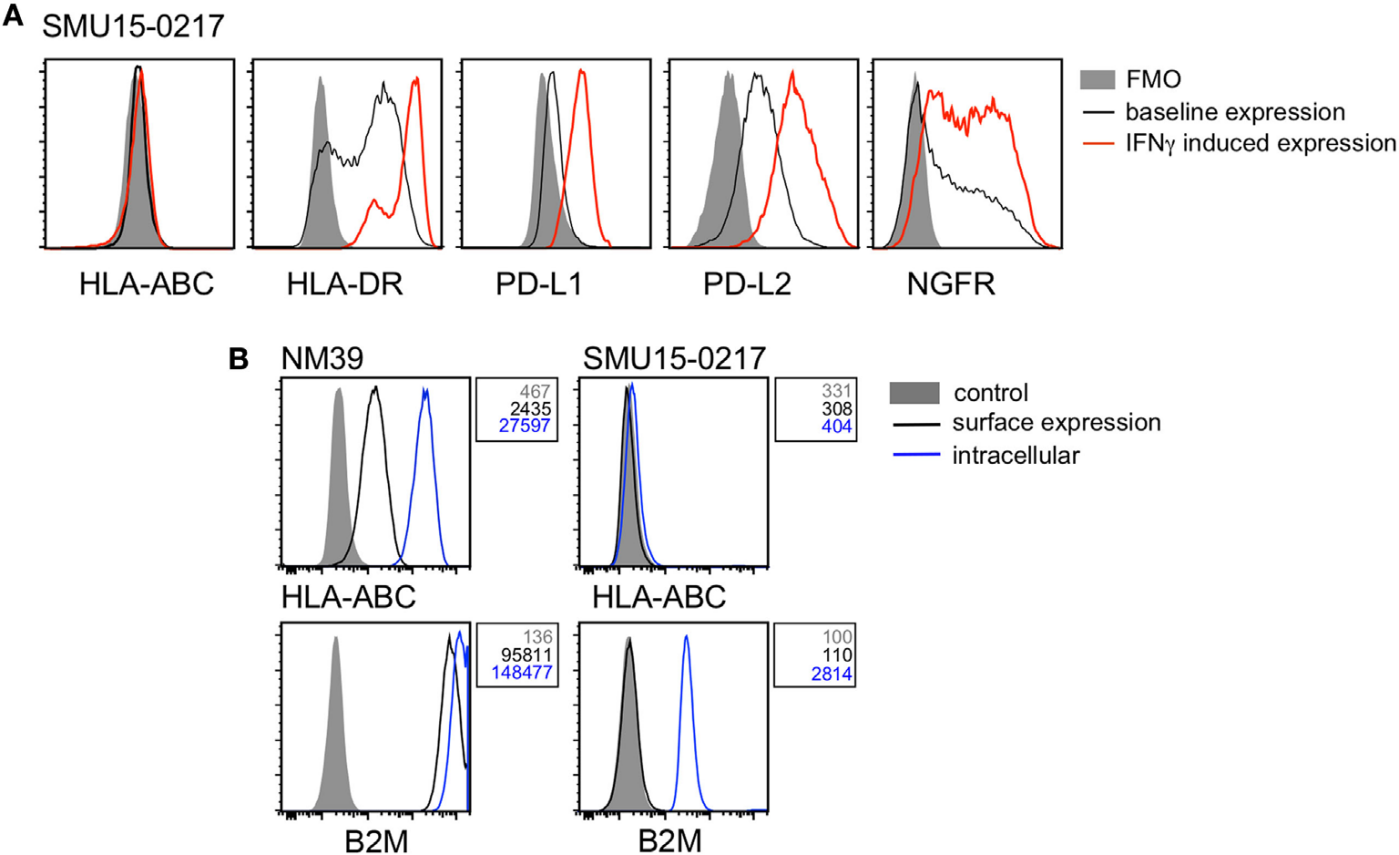
Analysis of β-2-microglobulin (B2M) expression in the SMU15-0217 cell line. **(A)** Representative histograms of cell surface expression of HLA-ABC, HLA-DR, PD-L1, PD-L2, and nerve growth factor receptor (NGFR) on SMU15-0217 cells. Baseline expression is shown in black, interferon-γ (IFNγ)-induced expression in red, and fluorescence minus one (FMO) controls as shaded histograms. **(B)** Expression of HLA-ABC and B2M on the cell surface (black) and intracellularly (blue) in NM39 and SMU15-0217 cells. Shaded histograms represent the mock stained control and mean fluorescence intensity values are shown next to the histograms. NM39 cells were used as a positive control.

**Figure 6:**
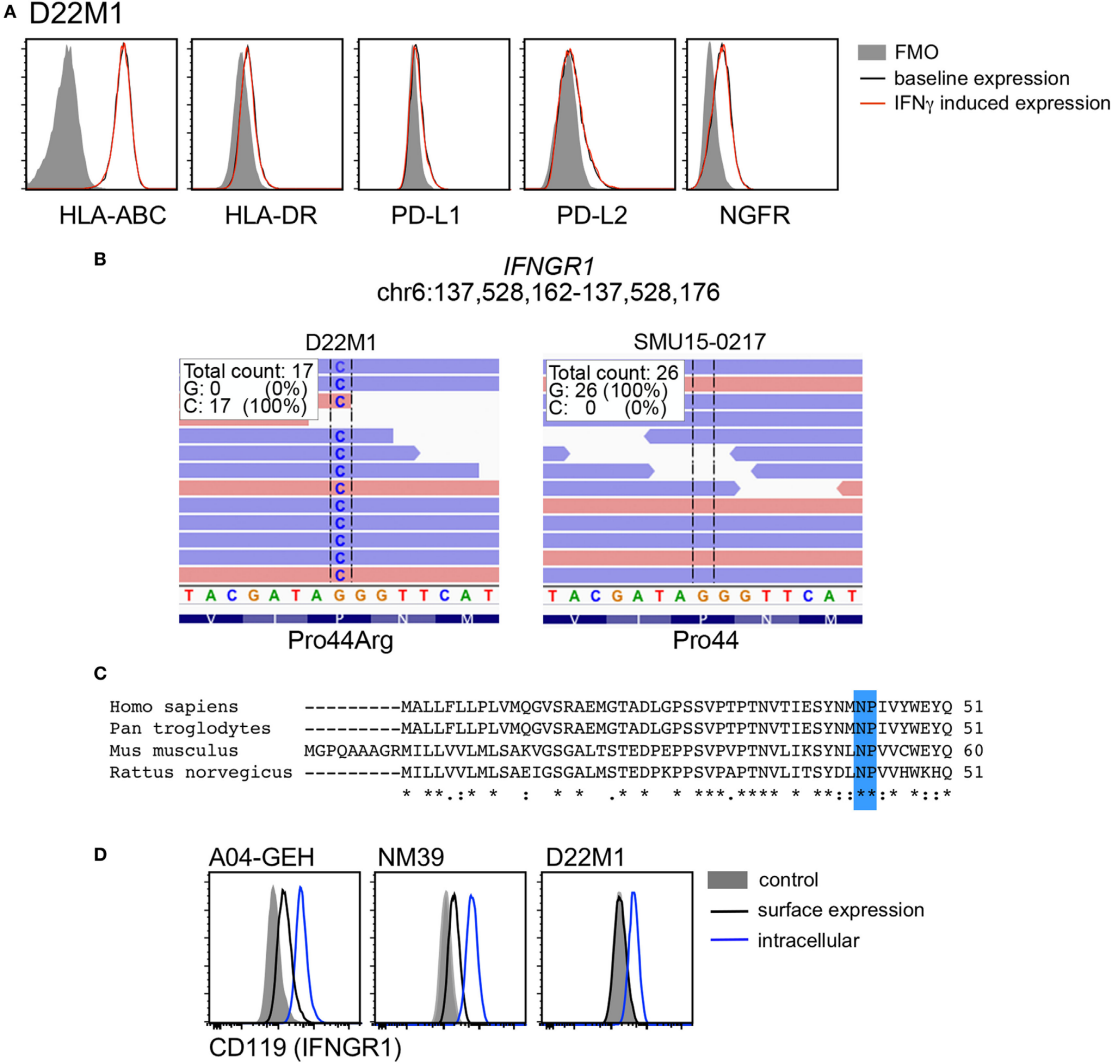
Analysis of interferon gamma receptor 1 (IFNGR1) expression in the D22M1 cell line. **(A)** Representative histograms of cell surface expression of HLA-ABC, HLA-DR, PD-L1, PD-L2, and nerve growth factor receptor (NGFR) on D22M1 cells. Baseline expression is shown in black, interferon-γ (IFNγ)-induced expression in red, and fluorescence minus one (FMO) controls as shaded histograms. **(B)** Whole exome sequencing analysis showing Pro44Arg (P44R) substitution in the D22M1 cell line but not in the SMU15-0217 cells. **(C)** Alignment of IFNGR1 protein sequence of human, chimpanzee, mouse, and rat (Clustal Omega) showing the highly conserved NP linker region highlighted in blue. **(D)** Expression of IFNGR1 on the cell surface (black) and intracellularly (blue) in A04-GEH, NM39, and D22M1 cells, with mean fluorescence intensity values also shown. Shaded histograms represent the mock stained control.

Twenty-six of 39 cell lines (67%) demonstrated diminished response to IFNγ stimulation, usually manifested as no induction (i.e., fold induction in MFI ratio < 1.5) of one or more markers in response to IFNγ stimulation. HLA-ABC expression was absent in the *BRAF/NRAS^WT^* SMU15-0217 cells even though expression of PD-L1, PD-L2, HLA-DR, and NGFR was upregulated by IFNγ (Figure 5A). Detailed analysis of this cell line confirmed that expression of B2M, the structural component of the MHC class I complex, was absent from the cell surface (Figure 5B). Among the other four markers, HLA-DR and PD-L1 expression was not induced in 7/39 cell lines, while induction of PD-L2 and NGFR was absent in 6/39 and 18/39 cell lines, respectively. One cell line, *BRAF/NRAS*^WT^ D22M1, showed a complete loss of response to IFNγ with no induction of any target molecules (Figure 6A), suggesting an upstream defect in the IFNγ signaling pathway in this cell line. Whole exome sequencing of this cell line identified a damaging missense mutation resulting in a P44R substitution in the extracellular portion of the IFNGR1 (Figure 6B). This amino acid substitution is located in the highly conserved NP linker region between the second and third beta sheets in the D1 domain (Figure 6C) and is classified as deleterious by the missense substitution algorithms SIFT and Polyphen-2 (data not shown). We confirmed that IFNGR1 expression was absent on the surface of D22M1 cells although IFNGR1 expression was detected intracellularly (Figure 6D), consistent with accumulation of a misfolded protein.

### Melanoma Cell Cycle Effects in Response to IFNγ Treatment

We also examined the impact of IFNγ treatment on cell cycle progression in our panel of melanoma cells using flow cytometry. Of the 38 melanoma cell lines tested, three showed increasing cell death in response to IFNγ, with greater than 10% increase in sub G1 (Table 2). Of these, one cell line (MM200) also showed a 56% increase in the proportion of cells undergoing DNA replication (i.e., S phase cells), along with another six cell lines that showed a greater than 30% increase in S phase cells. Another six cell lines, including 5/8 UVMs, showed diminished DNA replication post-IFNγ treatment (Table 2). The remaining 23 melanoma cell lines, including the IFNGR1-mutant D22M1 cells, showed minimal cell cycle profile changes when exposed to IFNγ (Table 2). It is worth noting that 5/7 melanoma cell lines with no IFNγ-mediated PD-L1 induction also showed no cell cycle profile changes in response to IFNγ treatment (Table 2).

**Table 2:** IFNγ-mediated cell cycle effects in melanoma cells.

Cell line	Driver mutation	Sub-G1 phase		G1 phase		S phase		G2 phase		
		−	+	−	+	−	+	–	+	IFNγ effect
A2058	BRAF^V600E^	0.6	1.3	68.2	63.3	19.2	26.7	12.7	10.1	↑ S phase
SKMel28	BRAF^V600E^	1.7	3.8	72.4	73.5	19.6	13.3	8.0	13.3	↓ S phase
C060M1	BRAF^V600E^	1.1	2.4	72.1	68.2	12.1	11.9	16.1	19.9	
SCC14-0257	BRAF^V600K^	1.6	5.3	63.4	50.5	20.0	26.1	16.6	23.4	↑ S phase
MM418	BRAF^V600E^	0.9	9.5	61.8	54.9	24.8	32.4	13.4	12.7	↑ S phase
NM16	BRAF^V600E^	0.8	4.1	65.6	70.1	26.3	26.8	8.1	3.1	
NM182	BRAF^V600E^	2.5	4.1	60.8	56.3	28.4	34.8	10.8	8.9	
MM200	BRAF^V600E^	1.1	17.1	69.9	62.2	19.9	31.1	10.2	6.7	↑ sub-G1, ↑ S phase
NM39	BRAF^V600E^	0.7	2.3	85.2	86.4	9.7	9.8	5.0	3.8	
HT144	BRAF^V600E^	1.6	12.9	65.4	61.7	24.3	24.4	10.4	13.9	↑ sub-G1
**C016M**	BRAF^V600E^	4.0	6.8	73.2	65.4	19.8	22.9	7.0	11.7	
MelRm	NRAS^Q61R^	0.7	4.1	62.9	64.9	26.8	24.2	10.3	10.8	
NM47	NRAS^Q61R^	0.4	6.5	63.0	65.3	25.8	24.3	11.2	10.4	
NM177	NRAS^Q61R^	2.6	2.9	67.6	59.5	23.3	28.4	9.0	12.2	
NM179	NRAS^Q61K^	1.7	6.7	60.1	52.5	24.8	32.9	15.0	14.7	↑ S phase
ME4405	NRAS^Q61R^	0.3	0.7	59.7	63.6	28.2	26.1	12.1	10.2	
MelAT	NRAS^Q61R^	0.5	0.8	58.2	68.0	29.7	22.7	12.2	9.3	
D11M2	NRAS^Q61L^	7.8	8.7	41.7	39.7	33.4	28.2	24.9	32.2	
C002M	NRAS^Q61K^	1.4	2.5	73.3	65.5	17.7	27.0	8.9	7.5	↑ S phase
C013M	NRAS^Q61L^	19.0	35.5	57.1	54.2	26.4	23.8	16.6	21.9	↑ sub-G1
D38M2	NRAS^Q61R^	0.7	1.3	65.1	59.9	18.2	21.3	16.7	18.9	
**D22M1**	BRAF/NRAS^WT^	1.2	1.2	52.7	50.9	37.9	39.5	9.4	9.5	
MeWo	BRAF/NRAS^WT^	1.1	1.7	49.2	52.9	25.5	24.1	25.4	22.9	
D24M	BRAF/NRAS^WT^	nd	nd	nd	nd	nd	nd	nd	nd	
**C022M1**	BRAF/NRAS^WT^	1.4	4.1	80.2	68.3	11.5	17.6	8.2	14.1	↑ S phase
C084M	BRAF/NRAS^WT^	0.6	1.2	37.0	37.8	22.5	16.4	40.6	45.8	
C086M	BRAF/NRAS^WT^	4.7	13.4	50.4	48.8	34.0	25.9	15.6	25.3	
D35M1	BRAF/NRAS^WT^	0.3	1.3	71.9	72.8	20.7	23.5	7.5	3.7	
C025M1	BRAF/NRAS^WT^	1.2	1.4	75.5	78.0	17.7	16.1	6.8	5.6	
SMU15-0217	BRAF/NRAS^WT^	0.6	1.4	69.5	67.5	22.2	21.0	8.3	11.5	
A04-GEH	BRAF/NRAS^WT^	1.0	7.7	60.0	56.9	25.7	24.7	14.3	18.4	
92.1	GNAQ^Q209L^	0.7	8.3	60.6	87.0	31.6	10.3	7.9	2.7	↓ S phase
MEL202	GNAQ^Q209L, R210K^	0.4	5.2	57.5	72.8	26.8	17.3	15.7	9.9	↓ S phase
**MEL270**	GNAQ^Q209P^	0.8	1.4	68.7	69.9	21.8	20.8	9.5	9.3	
MP38	GNAQ^Q209P^	0.6	2.2	72.7	88.4	12.0	4.2	15.4	7.4	↓ S phase
**OMM1**	GNA11^Q209L^	1.4	1.4	53.4	52.3	35.8	36.4	10.9	11.4	
**MP41**	GNA11^Q209L^	1.2	4.1	60.7	84.0	28.3	12.1	11.0	3.9	↓ S phase
**MP46**	GNAQ^Q209L^	1.2	1.8	28.7	29.1	10.4	10.1	57.3	61.4	
MM28	GNA11^Q209L^	0.7	1.2	85.6	92.4	7.1	3.4	7.3	4.3	↓ S phase

*Percentage of cells in the indicated cell cycle phase is shown. Data are the average of at least three independent experiments. S phase data indicate either 30% increase (↑) or decrease (↓) in the proportion of cells undergoing DNA replication, calculated as [(S phase_IFNγ_ − S phase_BSA_)/S phase_BSA_]*.

*↑ sub G1 indicates a greater than 10% increase in sub G1 cells in response to IFNγ treatment (sub G1_IFNγ_ − sub G1_BSA_)*.

*Cells showing no IFNγ-mediated PD-L1 induction are shown in bold*.

*−, no IFNγ treatment; +, treated for 72 h with 1,000 U/ml IFNγ; nd, not determined; IFNγ, interferon-γ; BSA, bovine serum albumin*.

## Discussion

Analysis of the IFNγ target proteins, HLA-ABC, HLA-DR, NGFR, PD-L1, and PD-L2, in a panel of 39 melanoma cell lines revealed that IFNγ stimulated cell surface expression of all five markers in only 13 melanoma cell lines tested. The degree of IFNγ-mediated induction was highly variable for all five markers but closely reflected the corresponding basal expression levels for HLA-ABC, HLA-DR, PD-L2, and NGFR. By contrast, PD-L1 expression was frequently absent at baseline (relative MFI < 1.5) but was still induced to high levels after IFNγ treatment in the majority of cell lines. Consequently, although the JAK/STAT/IRF1 pathway is critical for the IFNγ-mediated induction of HLA-ABC, HLA-DR, and the two PD-1 ligands (14, 23), the low constitutive expression of PD-L1 suggests that this pathway has low baseline activity in melanoma and that the constitutive expression of HLA-ABC, HLA-DR, PD-L2, and NGFR may be regulated *via* alternate pathways or downstream elements.

The IFNγ-induced expression of several markers, including PD-L1 and PD-L2, was correlated, although we did not detect significant correlation when the degree of IFNγ stimulation (i.e., change from pre- to post-stimulation) was compared. This may reflect disparate baseline expression levels due to IFNγ-independent regulatory influences but also the complexity and redundancy of the IFNγ signaling pathway. For instance, whereas the JAK–STAT1/2/3–IRF1 axis is critical for PD-L1 regulation, the JAK–STAT3–IRF1 node is important for PD-L2 stimulation (14). We also noted that cell surface expression of HLA-DR, NGFR, and PD-L2 was significantly lower in UVM compared to cutaneous melanoma, both at baseline and post-IFNγ stimulation. The transcriptomic analysis of the TCGA cutaneous and UVM datasets confirmed that UVM expressed lower levels of *HLA-DRA, NGFR, CD274* (PD-L1), and *PDCD1LG2* (PD-L2) transcripts, and this was associated with reduced transcript expression of the IFNγ master transcription factors *STAT1, STAT3*, and *IRF1* and with reduced IFNγ transcriptome signatures. It is worth noting that although transcriptome data are derived from high quality tumor samples with at least 60% tumor nuclei, they do contain variable levels of tissue-infiltrating immune and stromal cell populations that may influence the level of transcript expression (24). Nevertheless, collectively the transcriptome and flow cytometric analysis indicate diminished IFNγ activity in UVM.

Incomplete responses to IFNγ-stimulation, usually manifested as lack of induction of one or more markers were evident in 26 of 39 (67%) melanoma cell lines. Although it is still not clear whether incomplete IFNγ stimulation in melanoma cells has significant impact on patient responses to immunotherapy, it is evident that this pathway is important for response to PD-1 blockade. In particular, nuclear expression of the IFNγ transcription factor IRF1 (25) is associated with better response to anti-PD-1 therapy in melanoma (26) and loss-of-function mutations in IFNγ pathway modulators (JAK1, JAK2) are associated with resistance to anti-PD-1 treatment. Moreover, murine B16 melanoma cells deficient in JAK1 or IFNGR1 grew faster than control B16 cells in response to immune therapy (27). Metastatic UVM respond poorly to immune checkpoint inhibition (28, 29), and although there appears to be no difference in the level of infiltrating CD8+ T cells between uveal and cutaneous melanoma (30), our data suggest that UVM may have diminished capacity to respond to IFNγ, with lower expression of targets including PD-L1 (31), PD-L2, HLA-DR, and NGFR (this study). It is therefore provocative to suggest that inducibility of multiple IFNγ targets may inform or predict immunotherapy response.

It is worth noting that of the 26 melanoma cell lines displaying incomplete induction of the 5 target proteins, 8 showed cell cycle distribution changes in response to IFNγ treatment. Importantly, 5/7 melanoma cell lines with no IFNγ-mediated PD-L1 induction showed no cell cycle profile changes after treatment with IFNγ. This may reflect the critical role of the STAT1 transcription factor in promoting PD-L1 expression and mediating IFNγ-induced cell cycle effects (14, 32). Five of eight UVM cell lines responded to IFNγ treatment with a decreased proportion of S phase cells and this was not a common response in our panel of cutaneous melanoma cells. This may be due to IFNγ concentration effects, as previous reports have shown that 50 U/ml IFNγ was sufficient to arrest UVM cells, whereas concentrations exceeding 1,000 U/ml IFNγ were required to inhibit the growth of the cutaneous A375 melanoma cells (32, 33). The unique responses of UVM cells to IFNγ stimulation require further investigation.

Interestingly, although most of our cell lines did not display baseline PD-L1 expression, PD-L1 was induced in the majority of cell lines. This is significant, as PD-L1 expression is sufficient to mediate melanoma escape from immune checkpoint inhibition (34). Loss of MHC class I expression is another established mechanism of immune escape, often involving genetic alterations in the *B2M* gene (7, 13, 35) and we noted that the SMU15-0217 melanoma cell line showed loss of B2M expression, concurrent with loss of HLA-ABC expression. Only one cell line (i.e., D22M1) failed to respond to IFNγ, and this was associated with a homozygous, predicted loss-of-function mutation in the *IFNGR1* gene.

In conclusion, our study demonstrates that expression analysis of IFNγ targets pre- and post-IFNγ stimulation can identify incomplete IFNγ pathway activity in melanoma cells. We show that incomplete IFNγ signaling occurs in almost 70% of immunotherapy-naïve melanoma, and previous reports have confirmed that pre-existing alterations affecting IFNγ signaling have the potential to confer resistance to immune checkpoint inhibitors (7, 9). In fact, we identified two well-recognized mechanisms of immunotherapy resistance; the loss of B2M expression, resulting in absence of cell surface HLA-ABC, and a missense mutation in the *IFNGR1* gene, resulting in loss of cell surface IFNGR1. We also report that UVMs, which show poor responses to PD-1-inhibitor therapies, display an inherently weaker response to IFNγ signaling with reduced JAK–STAT pathway activity.

## Ethics Statement

This study was carried out in accordance with the recommendations of Human Research ethics committee protocols from Royal Prince Alfred Hospital (Protocol X15-0454 and HREC/11/RPAH/444) with written informed consent from all subjects. All subjects gave written informed consent in accordance with the Declaration of Helsinki. The protocol was approved by the Royal Prince Alfred Hospital Human Research ethics committee.

## Author Contributions

SA, ES, and AS performed the experiments. ES, HR, and SL wrote the manuscript. SA, SL, ES, and HR analyzed and interpreted the data. SA, AS, RK, SL, ES, and HR read, revised, and approved the final manuscript.

## Conflict of Interest Statement

RK served on advisory boards for Roche, Amgen, BMS, Merck, Novartis, and TEVA and has received honoraria from Merck, BMS, and Novartis. The remaining authors declare no conflict of interest.

## Supplementary Material

Figure S1 Flow cytometric analysis in melanoma cells. Representative histograms of baseline (solid black line) and IFNγ-induced expression (solid red line) of HLA-ABC, HLA-DR, NGFR, PD-L1, and PD-L2 in SKMel28 melanoma cells. Fluorescence minus one controls (FMO) are shown as shaded histograms.

Figure S2 PD-L1 and PD-L2 protein and transcript expression in melanoma cells. Correlation of cell surface protein [relative mean fluorescence intensity (MFI); left panel] and *CD274* (PD-L1) and *PDCD1LG2* (PD-L2) mRNA transcript expression derived from The Cancer Genome Atlas skin cutaneous melanoma dataset; right panel. Each dot represents one cell line. Spearman’s rank correlation coefficient and *p* values are shown.

Figure S3 Expression of downstream IFNγ targets post-IFNγ stimulation in cutaneous and uveal melanoma cells. Cell surface expression post-IFNγ stimulation (relative MFI) of HLA-ABC, HLA-DR, NGFR, PD-L1, and PD-L2 in cutaneous (*n* = 31) and uveal melanoma (*n* = 8) cell lines. Bars represent medians. Mann–Whitney test, *p* values are indicated.
