# Supplementary figures and images for: Interferon Signaling Is Frequently Downregulated in Melanoma
Source: Front Immunol. 2018 Jun 21;9:1414. doi: 10.3389/fimmu.2018.01414 (PMC6021492; doi:10.3389/fimmu.2018.01414)

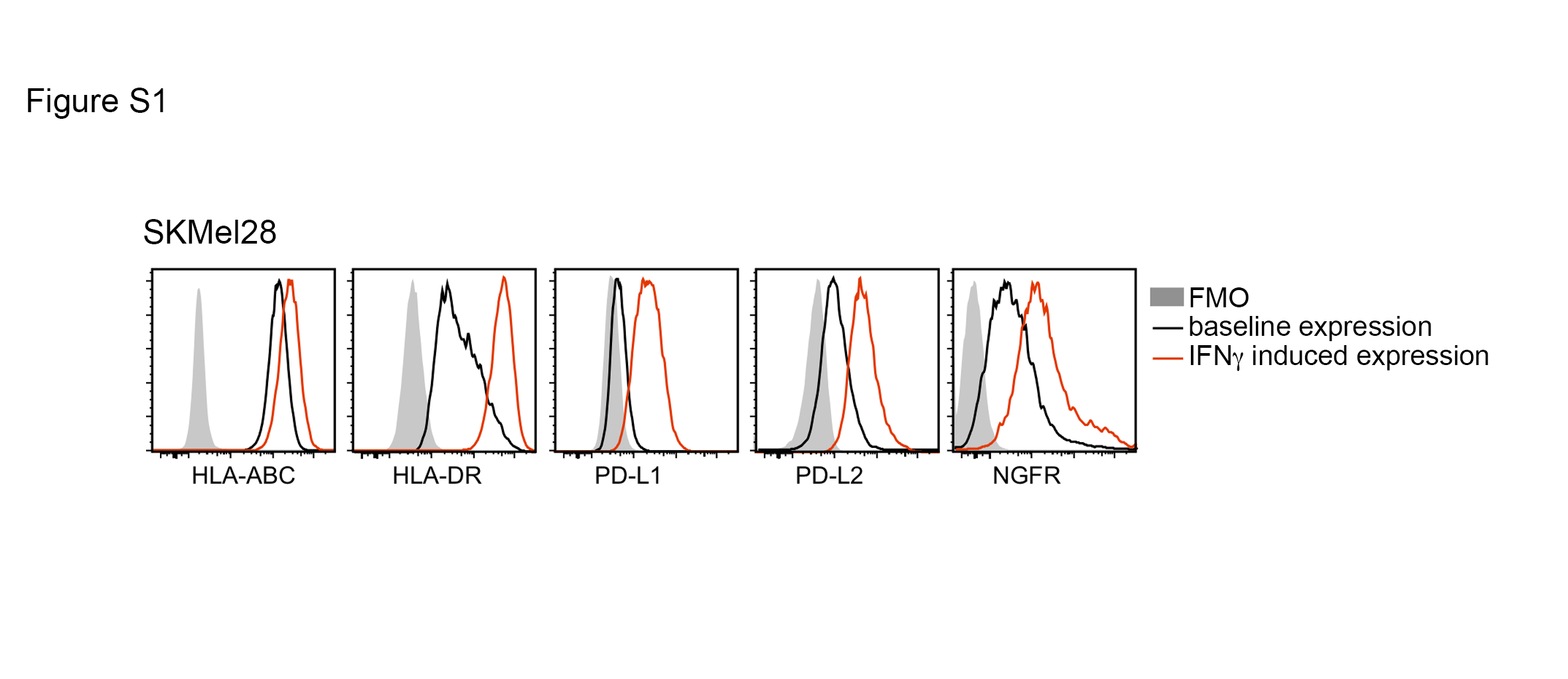

Supplement: Figure S1 — Flow cytometric analysis in melanoma cells. Representative histograms of baseline (solid black line) and IFNγ-induced expression (solid red line) of HLA-ABC, HLA-DR, NGFR, PD-L1, and PD-L2 in SKMel28 melanoma cells. Fluorescence minus one controls (FMO) are shown as shaded histograms. [file image_1.tif]

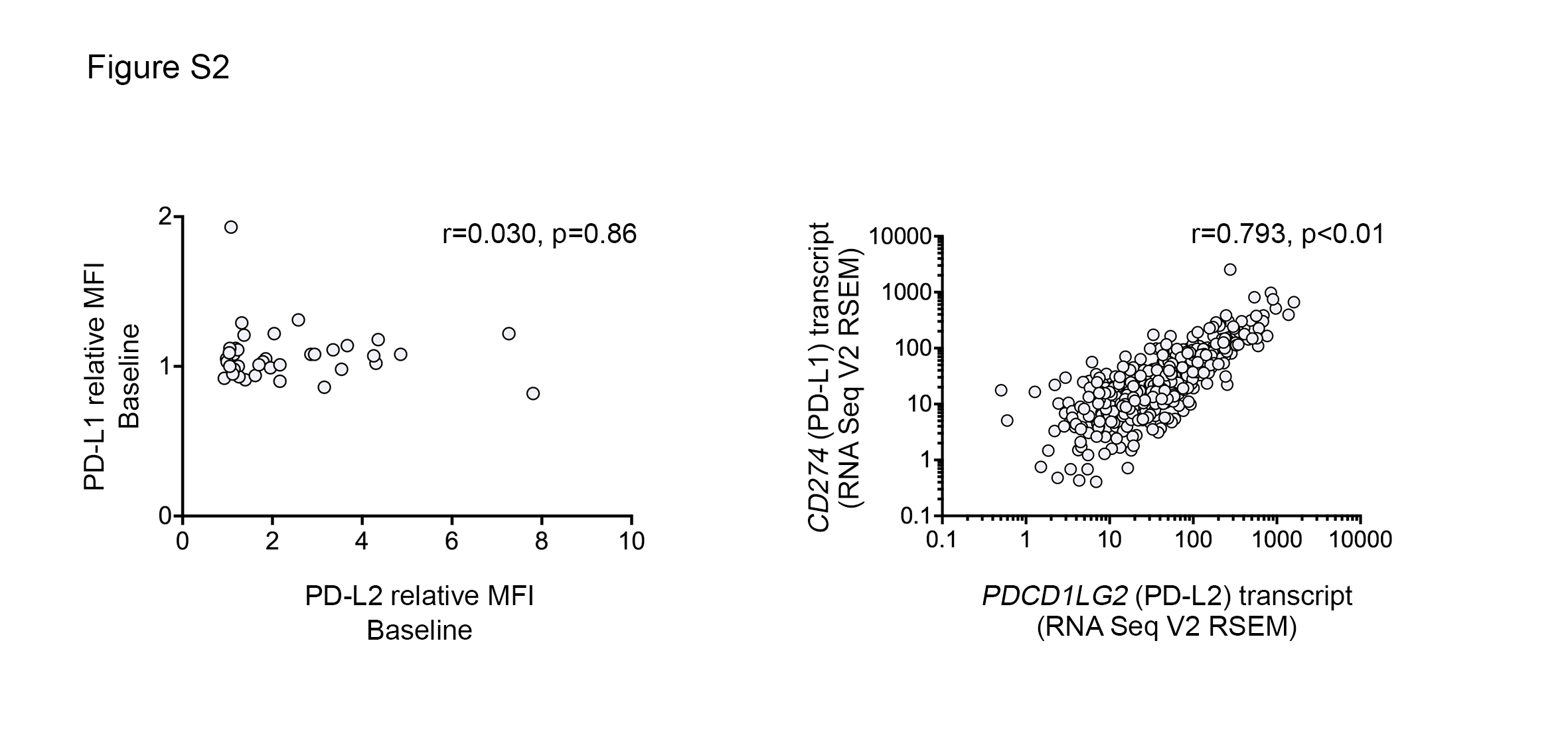

Supplement: Figure S2 — PD-L1 and PD-L2 protein and transcript expression in melanoma cells. Correlation of cell surface protein [relative mean fluorescence intensity (MFI); left panel] and CD274 (PD-L1) and PDCD1LG2 (PD-L2) mRNA transcript expression derived from The Cancer Genome Atlas skin cutaneous melanoma dataset; right panel. Each dot represents one cell line. Spearman’s rank correlation coefficient and p values are shown. [file image_2.tif]

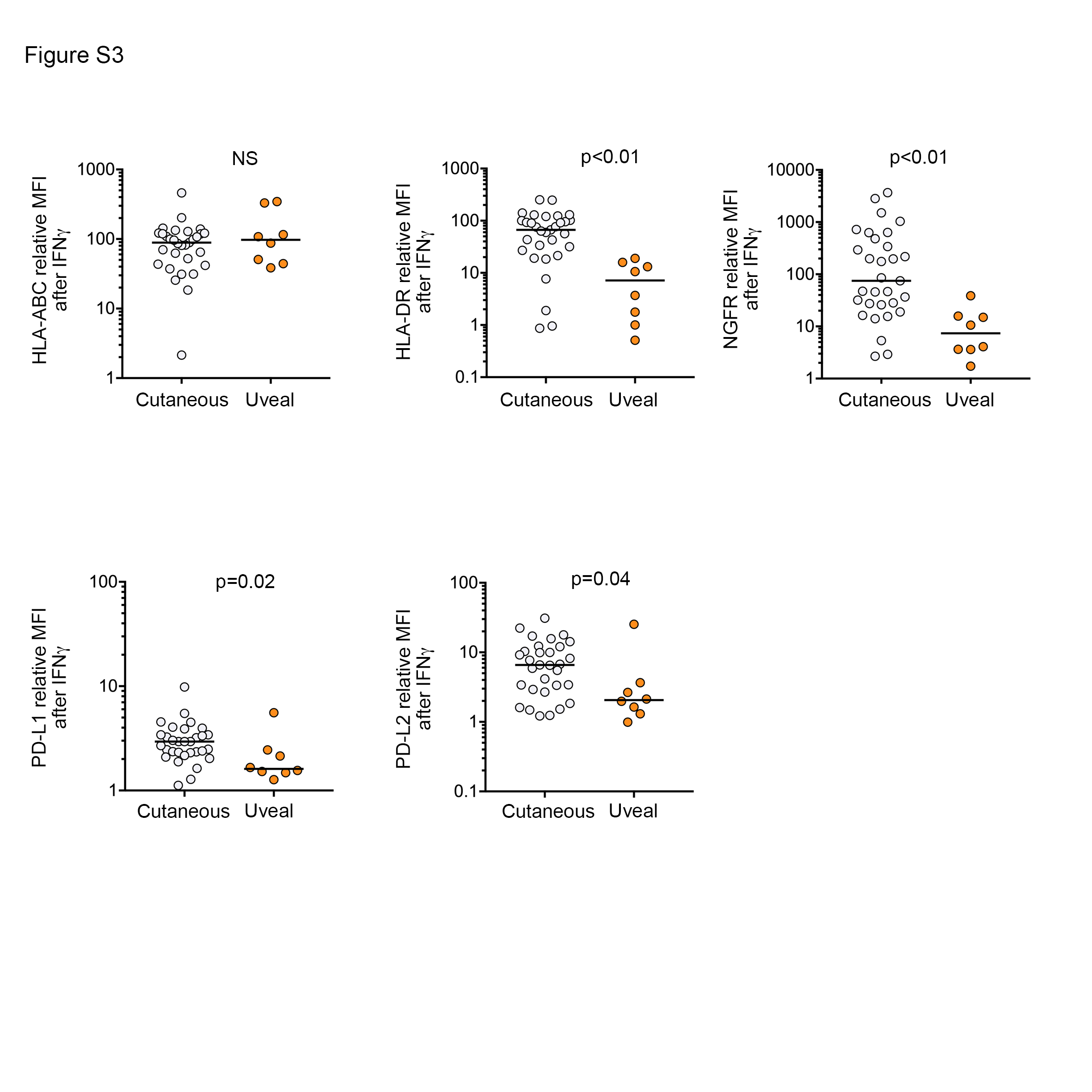

Supplement: Figure S3 — Expression of downstream IFNγ targets post-IFNγ stimulation in cutaneous and uveal melanoma cells. Cell surface expression post-IFNγ stimulation (relative MFI) of HLA-ABC, HLA-DR, NGFR, PD-L1, and PD-L2 in cutaneous (n = 31) and uveal melanoma (n = 8) cell lines. Bars represent medians. Mann–Whitney test, p values are indicated. [file image_3.tif]
